# Treatment with bortezomib for recurrent proliferative glomerulonephritis with monoclonal IgG deposits in kidney allograft. Case report and review of the literature

**DOI:** 10.1007/s40620-022-01332-x

**Published:** 2022-05-06

**Authors:** Rikako Oki, Kohei Unagami, Sekiko Taneda, Toshio Takagi, Hideki Ishida

**Affiliations:** 1 Department of Urology, Tokyo Women’s Medical University, 8-1 Kawatacho, Shinjuku-ku, Tokyo, 162-8666 Japan; 2 Department of Nephrology, Tokyo Women’s Medical University, Tokyo, Japan; 3 Department of Organ Transplant Medicine, Tokyo Women’s Medical University, Tokyo, Japan; 4 Department of Surgical Pathology, Tokyo Women’s Medical University, Tokyo, Japan

**Keywords:** Bortezomib, Kidney transplantation, Proliferative glomerulonephritis with monoclonal immunoglobulin, Recurrent glomerulonephritis

## Abstract

Proliferative glomerulonephritis with monoclonal immunoglobulin IgG deposits (PGNMID) is an already described form of renal involvement by monoclonal gammopathy. PGNMID is known to recur in kidney allografts. Bortezomib has shown clinical success in the treatment of multiple myeloma. However, its effect for recurrent PGNMID in kidney allografts has rarely been reported. We present the case of a 61-year-old woman who developed recurrent PGNMID 3 weeks after kidney transplantation. This patient was initially treated with steroid pulses (500 mg/day for 2 days) and two cycles of rituximab therapy (200 mg/body). However, disease progression was observed with mesangial matrix expansion and subendothelial deposits by light microscopy and stronger staining for IgG3 and kappa in the mesangial area by Immunofluorescence (IF) microscopy. Thus, we started treatment with bortezomib therapy (1.3 mg/m2, once weekly, on days 1, 8, 15, and 22 in a 5-week cycle, for a total of six cycles). Bortezomib therapy reduced massive proteinuria, although monoclonal immune deposits on IF and the serum creatinine level did not change during the treatment period. Seven months after completion of the first bortezomib course, we decided to prescribe a second course of bortezomib with the same regimen. Each course resulted in a > 50% reduction of proteinuria. Bortezomib may delay the progress of PGNMID in kidney allograft patients.

## Introduction

Proliferative glomerulonephritis with monoclonal immunoglobulin IgG deposits (PGNMID) is described as glomerulonephritis that is positive for the deposition of a single IgG subclass and a single light chain isotype [1]. Prognosis of this disease in the native kidney is unfavorable: 38% of patients recover renal function completely or partially, 38% develop persistent renal dysfunction, and 22% progress to end-stage renal disease (ESRD) [1]. Several reports have previously shown cases of recurrent PGNMID without a serum M spike after kidney transplantation (KT) [2, 3]. This suggests that persistent circulating factors in the recipient might be involved in the pathophysiology [2]. Most cases seem to have a low response rate to therapeutic strategies, including plasma exchange, high-dose prednisolone, rituximab, and bortezomib [2]. However, their actual effects on PGNMID remain obscure because of their low frequency. Wen et al. [3] reported three cases of recurrent PGNMID in renal allografts treated with bortezomib in their case series, but there was no detailed description of the clinical course, including pathological examination after administration of bortezomib. To date, there has been limited clinical experience regarding the use of bortezomib therapy for PGNMID in kidney allografts. Here, we report a patient with PGNMID after KT treated with two courses of bortezomib, who underwent several serial kidney biopsies.

## Case report

A 47-year-old woman who developed nephrotic syndrome with decreased kidney function was referred to our hospital for further investigation. At that time, kidney biopsy revealed mesangial expansion with positivity for IgG3, C3, C1q, and kappa (not lambda) on immunofluorescence (IF) staining. She was diagnosed with primary membranoproliferative glomerulonephritis (MPGN) because evidence of underlying diseases was lacking and the concept of PGNMID had not yet been established. Presently, at 61 years of age, she successfully underwent ABO-incompatible KT with her husband as a donor. Induction immunosuppressive therapy included tacrolimus, mycophenolate mofetil, methylprednisolone, and basiliximab. Rituximab (200 mg/body) and double filtration plasmapheresis were used for desensitization. A zero-hour biopsy revealed no abnormal findings. Her serum creatinine level remained within 1.1–1.2 mg/dl after KT.

### Biopsy 1 (3 weeks post-transplant) and treatment

**Fig. 1:**
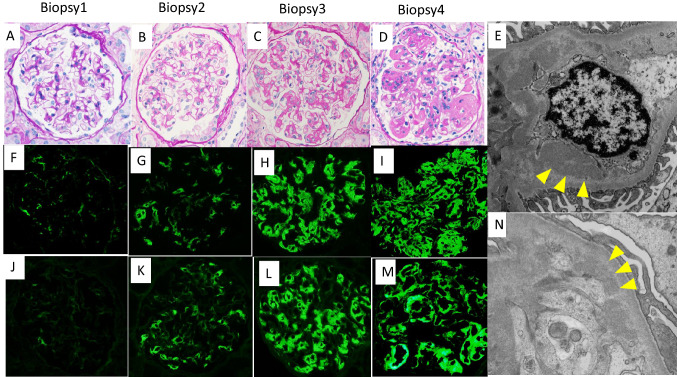
Kidney biopsy findings. **A** Findings of biopsy 1. The representative glomerulus shows no specific abnormality. **B** Findings of biopsy 2. The presented glomerulus is almost intact. **C** Findings of biopsy 3. Mesangial matrix expansion and segmental glomerular basement membrane thickening with a few double contours are observed in most of the glomeruli. **D** Findings of biopsy 4. Most of the glomeruli became lobulated. Mesangial matrix expansion together with mesangial cell proliferation and intracapillary proliferation are found. **E** Electron dense deposits in the mesangial area are observed in biopsy 1 (Yellow arrowheads show the deposits). **F**, **J** Slight granular mesangial staining for IgG3 (**F**) and kappa (**J**) is observed in the IF of biopsy 1. **G**, **K** Slight positive findings for IgG3 (**G**) and kappa (**K**) in the mesangial and para-mesangial area are observed in the IF of biopsy 2. **H**, **L** Stronger staining for IgG3 (**H**) and kappa (**L**) are found in the mesangial area and glomerular capillary walls in the IF of biopsy 3. **I**, **M** The positive region of IgG3 (**I**) and kappa (**M**) become more prominent in the immunofluorescence findings of biopsy 4. **N** Moderate amounts of mesangial deposits are observed in biopsy 2(Yellow arrowheads show the deposits). **A**–**D**: PAS staining × 400. PAS, Periodic Acid-Schiff

Three weeks after KT, she presented with abrupt worsening of graft function and her creatinine level rose to 1.42 mg/dl without urinary abnormalities. In the episode biopsy, there was no evidence of rejection (Fig. 1A). Slight granular mesangial staining for IgG3 and kappa (not lambda) in IF staining (Fig. 1F, J), and corresponding electron-dense deposits without any organized substructure in electron microscopy (EM) (Fig. 1E) were observed. The staining for C1q and C3 was weak to negative. Based on the above findings, the results of the native kidney biopsy could have been compatible with PGNMID when retrospectively reviewed. She was initially treated with half steroid pulse therapy (500 mg/day for 2 days), and serum creatinine stabilized to 1.1–1.2 mg/dl.

### Biopsy 2 (3 months post-transplant) and treatment

**Fig. 2:**
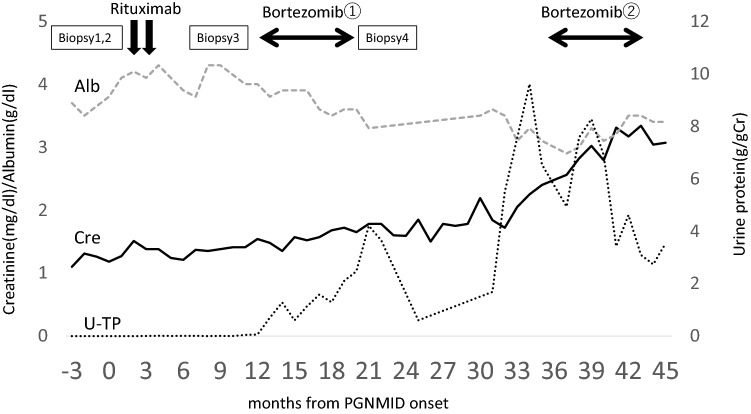
Clinical course of the disease. The serum creatinine level (mg/dl) and albumin level (g/dl) are shown in dotted and solid lines, respectively. The urinary protein level (g/gCre) is also indicated by a fine dotted line

A protocol biopsy at 3 months after KT showed no specific findings in light microscopy (Fig. 1B). Fibrosis in the cortical interstitial area was not evident. IgG3 deposition with kappa light chain restriction in the mesangial and para-mesangial areas was apparent in IF staining (Fig. 1G, K). Mesangial co-deposition of C3 and C1q was also observed. There were moderate amounts of mesangial deposits in the EM findings (Fig. 1N). Bone marrow biopsy excluded a lymphoproliferative disorder, and the serum kappa light chain was slightly elevated with a normal ratio of 1.38. The diagnosis of recurrent PGNMID in kidney allografts has become more robust, and based on these data she received two cycles of rituximab therapy (200 mg/body). Proteinuria remained negative, and serum creatinine level remained less than 1.5 mg/dl (Fig. 2).

### Biopsy 3 (11 months post-transplant) and treatment

Kidney biopsy was performed to evaluate the effectiveness of rituximab therapy 4 months after starting rituximab therapy. Light microscopy revealed mesangial matrix expansion and segmental glomerular basement membrane thickening with a few double contours in most of the glomeruli (Fig. 1C). Approximately 10% of the cortical interstitial area was fibrotic in Masson’s trichrome staining. Moreover, the IF findings showed stronger staining for IgG3, kappa (Fig. 1H, L), C1q, and C3 in the mesangial area and glomerular capillary walls, which reflected progressive disease. Thus, the decision was made to treat the patient with bortezomib (Velcade®) according to the multiple myeloma protocol at our hospital. Bortezomib was administered as a subcutaneous injection on days 1, 8, 15, and 22 in a 5-week cycle (1.3 mg/m^2^, once weekly) for a total of six cycles. Acyclovir was administered as a prophylactic medication to prevent varicella infection, which is one of the adverse events of bortezomib [3].

### Biopsy 4 (24 months post-transplant) and follow-up

The fourth biopsy was performed after completing bortezomib treatment. A vast extent of mesangial matrix expansion together with mesangial cell proliferation and intracapillary proliferation were observed, and the glomeruli became lobulated (Fig. 1D). The rate of fibrosis in the cortical interstitial area did not change in Masson’s trichrome staining. The positive regions of IgG3, kappa (Fig. 1I, M), C1q, and C3 became more prominent in the IF findings. Meanwhile, her urinary protein reached peak levels of 4.2 g/gCr, then decreased to 0.6 g/gCr 6 months after completing bortezomib therapy. Her serum creatinine level remained stable at less than 1.5 mg/dl (Fig. 2). Although pathological examination showed progressive disease, it is assumed that bortezomib has a certain positive clinical effect. However, seven months after the completion of bortezomib treatment, the patient was found to have proteinuria with protein excretion of 1.68 g/gCr and elevation of serum creatinine (1.78 mg/dl). Since worsening of kidney function (Cre 2.5 mg/dl) with massive urinary protein (9.6 g/gCr) was observed, we decided to administer a second course of bortezomib with the same regimen (Fig. 2). Urinary protein gradually resolved to 3.0 g/gCr with improvement in serum albumin during the treatment period. Serum creatinine level consistently stayed around 2.9–3.1 mg/dl at around the same time (Fig. 2).

## Discussion

We encountered a case of PGNMID that recurred in the allograft kidney within 3 weeks post-transplant, which was treated with 2 courses of bortezomib. Although the improvement of immunoglobulin deposits was not obvious in IF, reduction of proteinuria and elevation of serum albumin levels were observed after each bortezomib course. Therefore, we concluded that bortezomib has the potential to delay the progress of PGNMID in kidney allograft patients.

Although the pathophysiology of PGNMID remains unclear, hypersecretion of monoclonal IgG by clonal proliferation of B cells or plasma cells might be involved [1]. Monoclonal IgG molecules produced by B cell clones can self-aggregate and deposit in the glomeruli. In particular, IgG3 easily binds to C1q due to the relatively long hinge of IgG3, which leads to glomerular inflammation through complement activation [4, 5]. Steroids, rituximab, or antimyeloma agents (bortezomib or thalidomide) have been used empirically for PGNMID [4], but no standard treatment for PGNMID has been established.

Bortezomib is a proteasome inhibitor that prevents cell proliferation by inducing apoptosis in cancer cells [6]. Inhibition of the proteasome leads to prevention of the degradation of key proteins and cell death through multiple cascades [6]. This drug has shown great promise as a therapeutic agent for multiple myeloma [6]. In recent years, works have been published that support the role of bortezomib as a protective control for antibody-mediated rejection in KT recipients [7, 8]. Though little evidence has been presented, bortezomib induced a reduction in donor-specific anti-Human leukocyte antigen (HLA) antibody levels in some patients [7, 9]. This agent is assumed to inhibit autoantibody production by acting on plasma cells. Thus, bortezomib may be relatively easier to apply to KT patients.

The efficacy of bortezomib on PGNMID is still debatable because there are limited reports examining the effects of bortezomib in KT patients [3, 10]. Table 1 shows the previously published cases of recurrent PGNMID after KT treated with bortezomib. In their retrospective study, Said et al. reported that response to therapy (defined as > 50% reduction in proteinuria with < 25% increase in serum creatinine) was observed in 60% (9 of 15 patients with PGNMID in renal allograft, treated with immunosuppressive agents), two of whom were treated with a bortezomib-based regimen [11]. In our case, each bortezomib course resulted in a > 50% reduction in proteinuria. Bortezomib might play a role in the stabilization of renal function by reducing proteinuria, but renal dysfunction unfortunately progresses in the context of multiple factors, including age, interstitial fibrosis, tubular atrophy without any specific etiology (IF/TA), calcineurin inhibitor toxicity, and current glomerular injury.

**Table 1:** Previously published cases of recurrent PGNMID after kidney transplantation

Authors	Age, sex	Original disease of kidney dysfunction	Maintenance immunosuppressant	Duration from KT to onset	Treatment	Patient outcome	Follow up	Complication
Al-Rabadi et al. [13]	61/F	MPGN	TAC, MMF, steroids	98 months	bortezomib dexamethasone	Elevation of proteinuria	17 months	Not mentioned
Wen et al. [3]	30/F	Unknown	TAC, MMF, steroids	10.5 months	bortezomib, steroid pulse	ESRD	7 months	None
Wen et al. [3]	51/M	Unknown	TAC, MMF, steroids	11 months	bortezomib	Decreased proteinuria	11 months	Varicella infection
Wen et al. [3]	53/M	MPGN	TAC, MMF, steroids	33 months	bortezomib	Decreased proteinuria	1 month	None

M, male; F, female; MPGN, membranoproliferative glomerulonephritis; TAC, tacrolimus; MMF, mycophenolate mofetil; ESRD, end-stage renal disease

Our patient developed massive proteinuria 7 months after completing the first bortezomib course. It is interesting to note that the renal effect of bortezomib may persist for at least 6 months after therapy completion. At the time of writing, her proteinuria remained below 3 g/gCr (half of the peak proteinuria at the second flare) for 3 months after completing the second bortezomib course. Asthenic conditions (fatigue, malaise), gastrointestinal events (nausea, diarrhea, constipation, vomiting), and peripheral neuropathy are well recognized complications of bortezomib [6]. Bortezomib was significantly associated with a higher incidence of herpes zoster in the APEX trial comparing single-agent bortezomib with high-dose dexamethasone in MM patients [12]. Thus, we used acyclovir as a prophylactic medication for varicella infection. To date, our patient has not developed any adverse events related to bortezomib.

In conclusion, we report the detailed clinical course of recurrent PGNMID in a renal allograft patient, the progression of which may have been suppressed by two courses of bortezomib. The collection of similar cases is warranted to establish the best therapeutic strategy for PGNMID in KT patients.
